# catmap: Case-control And TDT Meta-Analysis Package

**DOI:** 10.1186/1471-2105-9-130

**Published:** 2008-02-28

**Authors:** Kristin K Nicodemus

**Affiliations:** 1 Genes, Cognition and Psychosis Program, Clinical Brain Disorders Branch, National Institute of Mental Health, National Institutes of Health, Bethesda, Maryland, USA; 2 Current address: Wellcome Trust Centre for Human Genetics, Roosevelt Drive, Oxford OX3 7BN, UK; 3 Department of Clinical Pharmacology, University of Oxford, Radcliffe Infirmary, Woodstock Road, Oxford, OX2 6HA, UK

## Abstract

**Background:**

Risk for complex disease is thought to be controlled by multiple genetic risk factors, each with small individual effects. Meta-analyses of several independent studies may be helpful to increase the ability to detect association when effect sizes are modest. Although many software options are available for meta-analysis of genetic case-control data, no currently available software implements the method described by Kazeem and Farrall (2005), which combines data from independent family-based and case-control studies.

**Results:**

I introduce the package catmap for the R statistical computing environment that implements fixed- and random-effects pooled estimates for case-control and transmission disequilibrium methods, allowing for the use of genetic association data across study types. In addition, catmap may be used to create forest and funnel plots and to perform sensitivity analysis and cumulative meta-analysis. catmap is available from the Comprehensive R Archive Network .

**Conclusion:**

catmap allows researchers to synthesize data to assess evidence for association in studies of genetic polymorphisms, facilitating the use of pooled data analyses which may increase power to detect moderate genetic associations.

## Background

Two study designs are commonly employed in genetic association studies: a case-control and a family-based approach. The case-control design compares frequencies of alleles carried among cases with a disease and among controls that are free of disease. The family-based design compares the frequency of alleles transmitted to an affected offspring by their parents with alleles carried by the parents but not passed to the offspring; this type of statistical analysis is often called a transmission disequilibrium test (TDT). Complex diseases are likely to be under the influence of several genetic risk factors; therefore, the contribution of a single gene to risk of disease is expected to be modest. Meta-analysis allows for the pooling of independent studies that examine similar hypotheses; for example, that a particular allele at a SNP is associated with disease status, and thus may improve power to detect moderate effect sizes. Although a few methods have been developed to combine family-based data with data from unrelated controls and/or unrelated cases obtained by the same study [1-10], most of these methods are not specifically designed to pool results from multiple independent studies and thus do not have a built-in test for heterogeneity of effect, which may be used to determine whether pooling of independent samples is reasonable. Exceptions to this statement include the method implemented in Genie[7,8], which conducts a genotype-based test that allows for the pooling of multiple study types across independent samples (meta-analysis), relying on empirical p-values which can be computationally intensive and SCOUT[5], which allows for the pooling of family-based data with data from unrelated cases and/or controls and performs a formal test of whether differing data types can be sensibly pooled. The likelihood-based method implemented in SCOUT[5] assumes that pooled samples are from the same source population with the same mating-type frequencies, and thus is inappropriate to use in a meta-analysis setting where independent samples have been drawn from differing populations; however, the use of additional programs such as the R package meta [10] subsequent to using SCOUT may be performed to pool estimates. A recent paper outlined a method for the combination of odds ratios (ORs) from independent case-control and TDT-based studies using a fixed-effect approach [6], using an allele-based model. Although several programs exist to conduct meta-analyses of case-control genetic data, no software exists that implements the Kazeem & Farrall (2005)[6] method to conduct case-control and TDT meta-analyses of independent samples. I have implemented this model [6], plus extended the method to the random-effects model of DerSimonian and Laird [11] in the freely-available R [12] package catmap.

## Implementation

Fixed-effect estimates are as described by Kazeem and Farrall [6]. First, ln(OR) estimates and their respective standard errors are derived from separate analyses for each independent study using traditional epidemiological methods for unmatched and matched case-control designs for 2 × 2 contingency tables (see [6] for review). Study-specific weights are derived using the inverse of the variance from each study (*w*_*i *_= 1/*σ*_i_^2^), which gives increased weight to larger studies (because their variances are generally smaller). The pooled estimate of the logarithm of the odds ratios using fixed effects methods (OR_FE_) is given by[6]:

$$\ln(OR_{FE})=\frac{\sum_{i=1}^{K}w_{i}\ln(OR_{i})}{\sum_{i=1}^{K}w_{i}}$$

with associated variance estimate of[6]:

$$\sigma_{\ln(OR_{FE})}^{2}=\frac{1}{\sum_{i=1}^{K}w_{i}}$$

where K is the number of studies to be pooled, and the *w*_*i *_are the study-specific weights as described above. In the presence of heterogeneity of genetic effects random-effects estimates of the pooled OR and variance may be more appropriate. Random-effects models do not assume that a common genetic effect exists across all samples. Following DerSimonian and Laird [11,13], the random-effects model implemented in catmap includes an estimate of the between study variance, *τ*^2^, as:

$$\tau^{2}=\frac{Q-(K-1)}{\sum_{i=1}^{K}w_{i}-\left(\frac{\sum_{i=1}^{K}w_{i}^{2}}{\sum_{i=1}^{K}w_{i}}\right)}$$

Where Q is the heterogeneity *χ*^2 ^statistic (see [6] for review), K is the number of studies and the *w*_i _are the study weights calculated using fixed-effects estimates as described above. If *τ*^2 ^≤ 0, *τ*^2 ^is set to 0 and random-effects estimates are identical to fixed-effects estimates. If *τ*^2 ^> 0 the random-effects weight for study *i *is *r*_*i *_= 1/(w_i_^-1 ^+ *τ*^2^) and the random-effects pooled estimate of the logarithm of the odds ratio (OR_RE_) can be written as:

$$\ln(OR_{RE})=\frac{\sum_{i=1}^{K}r_{i}\ln(OR_{i})}{\sum_{i=1}^{K}r_{i}}$$

and the standard error of the random-effects pooled ln(OR_RE_) is simply 1/(Σ*r*_*i*_)^1/2^.

## Results and Discussion

An example input file for catmap is shown in Table 1.

**Table 1:** 

name	study	t	nt	caserisk	controlrisk	casenotrisk	controlnotrisk
Yu,2004	1	78	21	NA	NA	NA	NA
Wu,2006	2	0	0	45	56	120	354

**Figure 1:**
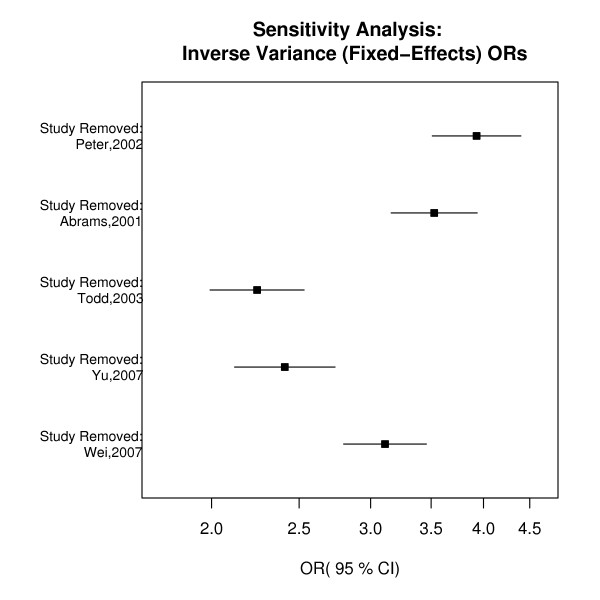
Sensitivity analysis plot created using test data set and function catmap.sense.

Input files are tab-delimited text files using the header shown in the example. The name of the first author and year of study, the type of study (1 = family-based, 2 = case-control) the number of alleles transmitted to affected offspring, the number of alleles not transmitted to affected offspring (both referring to family-based studies), the number of risk alleles in cases, the number of risk alleles observed in controls, the number of non-risk alleles observed in cases and the number of non-risk alleles observed in controls (latter 4 columns referring to case-control studies) must be specified. In the case of a family-based study, the **t **and **nt **columns should contain counts and the additional 4 columns may contain 0 or NA; for case-control studies, the columns **t **and **nt **should contain 0 or NA and the remaining 4 columns should contain the allele count data for cases and controls. catmap allows for the meta-analysis of all case-control or all family-based studies and for the combination of both family-based and case-control studies. In situations where both family-based and case-control data exist for the same set of cases only one type of study design (either family-based or case-control) should be used as input to catmap. catmap calculates fixed- and random-effects estimates, a *χ*^2 ^test for heterogeneity and associated p-values and must be used to create a catmap object for use in downstream functions. To create a forest plot using either the pooled fixed- or random-effects OR and 95% CI the function catmap.forest is used. catmap.sense calculates leave-one-out sensitivity analyses using either fixed- or random-effects estimates and creates a Portable Document Format (PDF) plot of the estimate of the pooled OR and 95% CIs with the removed study listed on the y-axis (Figure 1). Because the first report of a genetic association between a polymorphism and disease status often is larger than those reported by follow-up studies [14,15], the function catmap.cumulative may be used to calculate cumulative ORs and associated CIs and to create a PDF plot of the pooled estimates, adding one study at a time, using either fixed- or random-effects analyses. A graphic in PDF format to assess publication bias can be created using catmap.funnel.

## Conclusion

Pooling studies of genetic polymorphisms data via meta-analysis may improve the ability of researchers to detect modest association signals. Since case-control studies are not robust to spurious effects caused by confounding via population stratification, before combining results from case-control studies researchers should assess evidence for substructure in individual studies and also examine the similarity of effect sizes and allele frequencies across study type and population, along with the formal test of heterogeneity of genetic effect implemented in catmap. catmap implements a random-effects estimate of the pooled odds ratio, which assumes a Normal distribution of genetic effects across studies instead of a single genetic effect, which may be preferred when combining studies across sub-groups or when the heterogeneity *χ*^2 ^indicates significant between-study differences in effect sizes, although when evidence for heterogeneity exists the pooling of data may not be indicated. Since two types of study design (case-control and family-based) are common in statistical genetics, methods and freely-available software that can combine estimates from both designs should prove useful to applied researchers.

## Availability and Requirements

**Project name**: catmap

**Project home page**: Contributed package at .

**Operating systems**: MS Windows, Linux, Mac

**Programming language**: R

**Other requirements**: R 2.4 or higher

**License**: GPL

**Any restrictions to use by non-academics**: none

## Authors' contributions

KKN conceived of the random effects method, created the software and wrote the manuscript.

## References

[B1] Schaid DJ, Rowland C (1998). Use of parents, sibs, and unrelated controls for detection of associations between genetic markers and disease. Am J Hum Genet.

[B2] Becker T, Knapp M (2004). Maximum-likelihood estimation of haplotype frequencies in nuclear families. Genet Epidemiol.

[B3] Nagelkerke NJ, Hoebee B, Teunis P, Kimman TG (2004). Combining the transmission disequilibrium test and case-control methodology using generalized logistic regression. Eur J Hum Genet.

[B4] Becker T, Knapp M (2005). Impact of missing genotype data on Monte-Carlo simulation based haplotype analysis. Hum Hered.

[B5] Epstein MP, Veal CD, Trembath RC, Barker JNWN, Li C, Satten GA (2005). Genetic association analysis using data from triads and unrelated subjects. Am J Hum Genet.

[B6] Kazeem GR, Farrall M (2005). Integrating case-control and TDT studies. Ann Hum Genet.

[B7] Allen-Brady K, Wong J, Camp NJ (2006). PedGenie: an analysis approach for genetic association testing in extended pedigrees and genealogies of arbitrary size. BMC Bioinformatics.

[B8] Curtin K, Wong J, Allen-Brady K, Camp NJ (2007). PedGenie: meta genetic association testing in mixed family and case-control designs. BMC Bioinformatics.

[B9] Dudbridge F UNPHASED user guide. Technical Report 2006/5.

[B10] Schwarzer G The meta Package. [R package version 08-2].

[B11] DerSimonian R, Laird N (1986). Meta-analysis in clinical trials. Control Clin Trials.

[B12] R Development Core Team (2007). R: A Language and Environment for Statistical Computing.

[B13] Egger M, Davey Smith G, Altman DG (2001). Systematic reviews in health care: Meta-analysis in context.

[B14] Ioannidis JP, Ntzani EE, Trikalinos TA, Contopoulos-Ioannidis DG (2001). Replication validity of genetic association studies. Nat Genet.

[B15] Trikalinos TA, Ntzani EE, Contopoulos-Ioannidis DG, Ioannidis JP (2004). Establishment of genetic associations for complex diseases is independent of early study findings. Eur J Hum Genet.

